# The phosphoinositide 3-kinase signaling pathway in normal and malignant B cells: activation mechanisms, regulation and impact on cellular functions

**DOI:** 10.3389/fimmu.2012.00224

**Published:** 2012-08-09

**Authors:** Samantha D. Pauls, Sandrine T. Lafarge, Ivan Landego, Tingting Zhang, Aaron J. Marshall

**Affiliations:** ^1^Department of Immunology, University of Manitoba,Winnipeg, MB, Canada; ^2^Department of Biochemistry and Medical Genetics, University of Manitoba,Winnipeg, MB, Canada; ^3^Manitoba Institute for Cell Biology, CancerCare Manitoba,Canada; ^4^Yale University,USA

**Keywords:** antibody, antigen receptor, B lymphocyte, germinal center, inositol phosphatase, isotype switch, leukemia and lymphoma, phosphatidylinositol 3-kinase

## Abstract

The phosphoinositide 3-kinase (PI3K) pathway is a central signal transduction axis controlling normal B cell homeostasis and activation in humoral immunity. The p110δ PI3K catalytic subunit has emerged as a critical mediator of multiple B cell functions. The activity of this pathway is regulated at multiple levels, with inositol phosphatases PTEN and SHIP both playing critical roles. When deregulated, the PI3K pathway can contribute to B cell malignancies and autoantibody production. This review summarizes current knowledge on key mechanisms that activate and regulate the PI3K pathway and influence normal B cell functional responses including the development of B cell subsets, antigen presentation, immunoglobulin isotype switch, germinal center responses, and maintenance of B cell anergy. We also discuss PI3K pathway alterations reported in select B cell malignancies and highlight studies indicating the functional significance of this pathway in malignant B cell survival and growth within tissue microenvironments. Finally, we comment on early clinical trial results, which support PI3K inhibition as a promising treatment of chronic lymphocytic leukemia.

## INTRODUCTION: THE PHOSPHOINOSITIDE 3-KINASE PATHWAY IN LYMPHOCYTES

**FIGURE 1:**
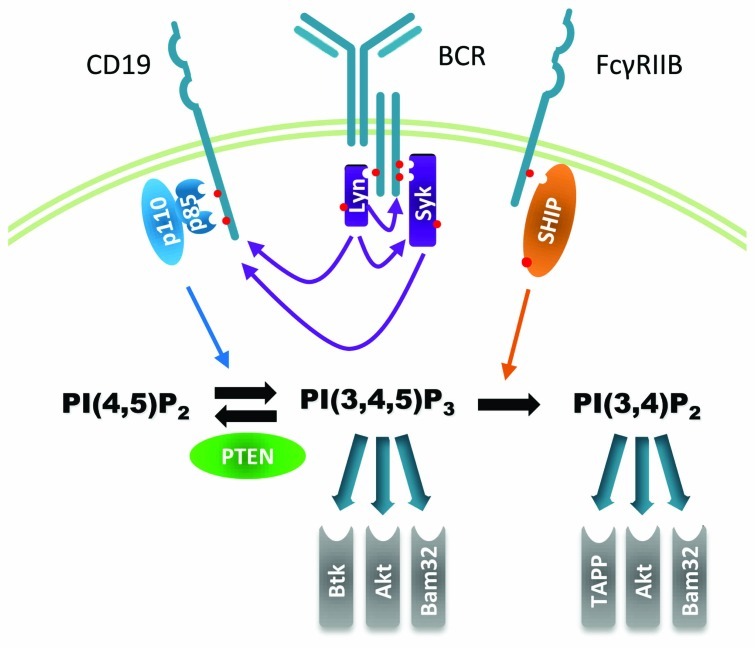
**Major components of the PI3K pathway in B cells discussed in this review**. PI3K pathway activation via B cell antigen receptor is depicted schematically. Lyn and Syk are recruited to the engaged BCR where they are activated by phosphorylation and initiate the downstream signaling cascade. Class I PI3K such as p110δ or p110α binds phosphorylated CD19 and catalyzes the production of PIP_3_. Alternative PI3K recruitment mechanisms not shown include binding to BCAP, TC21, or IRS-2/IL-4R. Several PH domain-containing signaling proteins bind to PIP_3_ including enzymes such as Btk and Akt and adaptor proteins such as Bam32. PIP_3_ levels are down-regulated by phosphatases PTEN and SHIP. PTEN directly opposes the PI3K reaction while SHIP, classically activated downstream of FcγRIIB co-aggregation with the BCR, produces a novel second messenger PI(3,4)P_2_ which can recruit its own set of PH domain proteins. Recruited PI-binding proteins are enzymatically activated and/or serve to form membrane-associated protein complexes that regulate downstream signaling and cytoskeletal rearrangement.

Phosphoinositide 3-kinases (PI3K) are a family of enzymes that selectively phosphorylate the D3 hydroxyl group of the inositol head group of phosphoinositide (PI) lipids. PI3K enzymes can be divided into three families based on their structure and PI specificity. Class I PI3K enzymes are heterodimeric complexes composed of regulatory (p85α, p85β, p55γ, p55α, p50α, and p101) and catalytic (p110α, p110β, p110δ, or p110γ) subunits. Class I PI3K enzymes can generate two major species of D3 phosphoinositides, PI(3,4,5)P_3_ and PI(3,4)P_2_. Class II PI3K enzymes selectively phosphorylate PI and PI(4)P to produce PI(3)P and PI(3,4)P_2_, whereas class III only produce PI(3)P (Deane and Fruman, 2004). Little is known about the role of class II and III PI3Ks in lymphocytes and they will not be reviewed here. The accumulation of PI(3,4,5)P_3_ and PI(3,4)P_2_ are further controlled by PI phosphatases, including phosphatase and tensin homolog (PTEN) and SH2 domain-containing inositol-5′-phosphatase 1 (SHIP1 or SHIP). Specific binding of intracellular signaling enzymes and scaffold proteins to PI(3,4,5)P_3_ and PI(3,4)P_2_ are thought to mediate the functional outcomes triggered by these second messengers molecules. Collectively we refer to PI3K enzymes, PI phosphatases, D3 phosphoinositides, and their direct binding partners as the PI3K pathway (**Figure 1**). The cellular responses triggered by the PI3K pathway are highly influenced by integration with other signals that can directly impact PI-binding proteins and also modify downstream events.

Substantial evidence now exists indicating that development and activation of B lymphocytes is highly dependent on the PI3K pathway. Mature B cell survival and maintenance requires continuous expression of a signaling-competent B cell antigen receptor (BCR) which is thought to support a basal level of “tonic” signaling (Lam et al., 1997; Tze et al., 2005). Enforced PI3K activity was found to promote survival of mature B cells in the absence of BCR expression (Srinivasan et al., 2009). Abundant evidence has emerged indicating that activation of the PI3K pathway is required for B cell survival and differentiation into mature B cell subsets, as well as a number of mature B cell immune functions. In addition to functions in normal B cells, several types of B cell malignancy show evidence of deregulation of the PI3K pathway. Moreover, some malignant B cells appear to be dependent on this pathway for survival and retention in protective lymphoid tissue niches.

The functional and potential clinical importance of this pathway continues to drive research into molecular regulation of this pathway in B cells. While many components of are conserved among cell types, lymphocytes appear to have a few unique modifications of the core pathway. While the p110α and β catalytic subunits are ubiquitously expressed, the p110δ and p110γ are largely restricted to leukocytes (Chantry et al., 1997; Vanhaesebroeck et al., 1997). Class IA PI3K enzymes such as p110δ are classically thought to be activated by tyrosine kinase-linked receptors whereas class 1B PI3K (p110γ) is activated through G protein-coupled receptors (Deane and Fruman, 2004). Consistent with this model, the weight of evidence suggests that p110γ is not essential for B cell signaling via antigen (Ag) and cytokine receptors (Reif et al., 2004; Beer-Hammer et al., 2010). However, a more complex picture has emerged regarding p110δ which seems to have some involvement in signaling via G protein-coupled chemokine receptors. Immune cells also selectively express the PI phosphatase SHIP1, and some PI-binding proteins such as Bam32/DAPP1 and Btk. These molecules appear to have regulatory functions specific to immune cells and clearly function in B cells. The goal of this review is to bring together for discussion the current evidence on molecular regulation and cellular functions of the PI3K pathway in the context of normal and malignant B cells.

## MECHANISMS OF PI3K PATHWAY ACTIVATION IN NORMAL B CELLS

### CONTROL OF PI3K ENZYMATIC ACTIVITY

Class IA PI3Ks at rest exist predominantly in an inhibited conformation. The p110 catalytic subunit constitutively associates with the regulatory subunit via its interstitial SH2 domain (iSH2). This iSH2 bridges two other SH2 domains, the N-terminal and C-terminal SH2 domains (nSH2 and cSH2, respectively), which also associate weakly with p110 and promote an inhibited conformation (Yu et al., 1998b). Activation of catalytic activity involves binding of nSH2 and cSH2 to phosphopeptide motifs such as pTyr-x-x-Met. This binding both recruits the complex to the membrane and dissociates these SH2 domains from p110, relieving their inhibitory effect (Carpenter et al., 1993; Yu et al., 1998b). Intriguingly, the precise mechanism of regulation by p85 may differ between p110 isoforms (Burke et al., 2011). It was previously shown for p110α that the minimal p85 domains required for inhibition are the nSH2 plus iSH2, where nSH2 is responsible for reversible inhibition in the absence of phosphopeptide binding (Yu et al., 1998a). More recently, it has been demonstrated that p110δ and p110β have a distinct mechanism of inhibition involving cSH2 in addition to nSH2 (Burke et al., 2011; Zhang et al., 2011), suggesting differential regulation of these isoforms by cSH2-binding phosphopeptide ligands. Recent evidence suggests that binding of p110 itself to anionic lipids in the cell membrane contributes to enhancement of catalytic activity for all Class IA isoforms. Access to these lipids is regulated by p85 and, at least for p110α, seems to confer a global conformational change (Hon et al., 2011). Binding of the catalytic subunit to certain Ras family GTPases at the membrane also contributes to activation (Rodriguez-Viciana et al., 1994, 2004; Jimenez et al., 2002). Interestingly, p110 isoforms show selectivity in activation by distinct Ras family members, with p110δ showing selective activation by R-Ras1 and R-Ras2 (Rodriguez-Viciana et al., 2004). TC21/R-Ras2 has recently been shown to act by mediating an interaction between the BCR and both p110δ and p85α, thereby recruiting P13K to the cell membrane (Delgado et al., 2009).

Phosphoinositide 3-kinase is activated downstream of BCR ligation in a manner dependent on the src family kinase Lyn and sustained by the tyrosine kinase Syk (Kurosaki et al., 1994; Beitz et al., 1999). Ag binding to the BCR induces tyrosine phosphorylation of the cytoplasmic tails of the associated signaling chains, Ig-α and Ig-β by Lyn and Syk (Kurosaki et al., 1994; Beitz et al., 1999). It has been proposed that both a conformational change in the receptor and a change in the local lipid environment may be required for this signal initiation step (Tolar et al., 2005). Lyn and Syk also phosphorylate CD19 (Tuveson et al., 1993; Fujimoto et al., 2000) and B cell adaptor protein BCAP (Okada et al., 2000; Yamazaki et al., 2002; Aiba et al., 2008). These both co-aggregate with the BCR complex in the cell membrane and directly bind p85 via YxxM motifs. CD19 and BCAP are reported to make complementary and functionally important contributions to PI3K activation in B cells (Aiba et al., 2008), with CD19 ligation via the complement receptor functioning to reduce the threshold of Ag required to trigger B cell activation (Carter and Fearon, 1992; Buhl et al., 1997; Wang et al., 2002). The guanine nucleotide exchange factor Vav is required for PI3K pathway activation downstream of BCR–CD19 co-ligation but not BCR ligation alone (Vigorito et al., 2004). Recent studies suggest the adaptor protein Grb2 is required for efficient PI3K signaling in B cells, however the mechanism is not yet clear (Ackermann et al., 2011).

Receptors other than the BCR can also induce PI3K signaling in B cells. PI3K is clearly activated by IL-4R and p110δ is required for functional responses to IL-4 (Bilancio et al., 2006). PI3K recruitment and activation downstream of the IL-4 receptor seems to be mediated primarily by insulin receptor substrate (Zamorano et al., 1996). Activation downstream of the IL-3 receptor has been shown in some systems to involve binding of p85 to the adaptor protein Gab2 which localizes to activated cytokine receptors via a Shc–Grb2–Gab2 complex (Gu et al., 2000). Activity downstream of Toll-like receptors has also been reported and seems to be mediated by BCAP (Ni et al., 2012; Troutman et al., 2012). PI3K activation downstream of CD40 ligation depends on casitas B-lineage lymphoma (c-CBL), acting as an adaptor protein (Arron et al., 2001). B cell activating factor (BAFF) binding to its receptor on B cells is reported to induce p110δ activation (Patke et al., 2006; Henley et al., 2008), however the mechanism is yet to be characterized.

### CONTROL OF PI PHOSPHATASE ACTIVITY

PTEN activity is regulated by a variety of mechanisms, including interactions with lipids and proteins, regulation of protein levels, serine/threonine phosphorylation, and catalytic inactivation by reactive oxygen species (ROS). *In vitro* studies revealed that dynamic binding of the N-terminus of PTEN to anionic lipids, especially the PTEN substrate PI(4,5)P_2_, leads to a conformational change and an increase in PTEN phosphatase activity (McConnachie et al., 2003). PTEN protein is sensitive to regulation by ubiquitination followed by proteasomal degradation (Wang et al., 2007). Interestingly, monoubiquitination has also been reported and was shown to promote nuclear import (Trotman et al., 2007). PTEN levels can also be controlled post-transcriptionally by the microRNA cluster miR-17-92 (Rao et al., 2011). Serine/threonine phosphorylation appears to be a double edged sword in terms of PTEN regulation, with the outcome depending on the precise site. Deletion of the C-terminal tail of PTEN, which contains several phosphorylation sites, revealed a role both in dampening catalytic activity and increasing protein stability (Vazquez et al., 2000). The effect on catalytic activity was later proposed to be the result of a conformational change induced by phosphorylation (Vazquez et al., 2001; Odriozola et al., 2007), while the effect on stability is due to protection from proteasomal degradation (Torres and Pulido, 2001). A study in Jurkat T cells provided evidence for a feedback loop involving phosphorylation of Thr366 by GSKβ, a downstream effector of PI3K, which was reported to inhibit PTEN activity (Al-Khouri et al., 2005). Thr366 phosphorylation was also found to decrease PTEN stability in glioblastoma cell lines (Maccario et al., 2007). One particularly interesting PTEN-binding protein that seems to directly promote its enzymatic activity is p85α, the regulatory subunit classically associated with the PI3K p110 subunit (Taniguchi et al., 2006; Chagpar et al., 2010).

A number of reports show that PTEN activity can be regulated by ROS. Specifically, oxidation by either exogenous or endogenous H_2_O_2_ leads to the formation of a disulfide bond between cysteine 124, found in the active site, and cysteine 71 (Lee et al., 2002). ROS can be produced in B cells by NADPH oxidase activity triggered downstream of various receptors (Hancock et al., 1990; Lee and Koretzky, 1998). Our group demonstrated that treatment of B cell lines with H_2_O_2_ lead to selective accumulation of PI(3,4)P_3_-specific pleckstrin homology (PH) domains at the cell membrane. A synergistic effect was observed with co-stimulation through the BCR. This is consistent with a role for H_2_O_2_ in the catalytic inactivation of PTEN but not SHIP (Cheung et al., 2007). Since hydrogen peroxide has been proposed as a significant “second messenger” for B cell activation (Reth, 2002), and ROS production by neutrophils and macrophages is a ubiquitous component of inflammation, oxidative inactivation of PTEN may be an important mechanism contributing to PI3K pathway activation in infectious disease and chronic inflammatory disease. All of these regulatory mechanisms, including binding to lipid and protein partners, degradation, post-transcriptional repression, Ser/Thr phosphorylation, and inactivation by ROS, have been described; however their relative importance in B cells remains to be determined.

SHIP phosphatase activity is regulated at the levels of expression, sub-cellular localization, phosphorylation, and conformation. Expression levels can be altered either by translational inhibition mediated by the microRNA miR-155 (Costinean et al., 2009) or by ubiquitin-mediated proteasomal degradation (Ruschmann et al., 2010). The principal activation mechanism of SHIP catalytic function seems to be recruitment to the cell membrane where it can access its substrate, PI(3,4,5)P_3_ (Phee et al., 2000). Classically, this is accomplished by binding of its SH2 domain to phosphorylated immunoreceptor tyrosine-based inhibitory motifs (ITIMs) in the cytoplasmic tail of the inhibitory receptor FcγRIIB when this receptor is co-engaged with the BCR (Tridandapani et al., 1997). In mice, another C-terminal tyrosine residue outside the ITIM in FcγRIIB is reported to be required for SHIP binding as it allows the formation of a stabilized tri-molecular complex that includes Grb2 and/or Grap (Isnardi et al., 2004). In human FcγRIIB, the corresponding C-terminal sequence contains no tyrosine residue, however the segment itself is required for SHIP binding (Isnardi et al., 2006). These studies support earlier reports that the C-terminus of SHIP, containing both a proline rich region as well as several phosphorylation sites, is required in addition to the minimal phosphatase domain to efficiently mediate downstream inhibition of Ca^2+^ flux (Aman et al., 2000).

Although SHIP mediates the inhibitory functions of FcγRIIB in B cells (Isnardi et al., 2006), SHIP can also act independently of FcγRIIB to regulate signaling via the BCR and other receptors (Brauweiler et al., 2000). In many cases, the proteins responsible for recruitment of SHIP to the membrane are uncharacterized, however there are reports that SHIP can bind ITAMs in receptors such as FcγRIIA (Maresco et al., 1999), FcεRI (Kimura et al., 1997), and perhaps the Ig-α chain of the BCR under some conditions (Mukherjee et al., 2012). Once recruited to the membrane, SHIP is phosphorylated on tyrosine residues in its C-terminus by Lyn kinase (Tridandapani et al., 1997; Phee et al., 2000). Although the C-terminal segment is required for full enzymatic activation (Aman et al., 2000) and tyrosine phosphorylation, especially at Tyr1020 has been frequently used as a functional readout, it seems that it does not directly promote 5′-phosphatase activity (Phee et al., 2000). The main function of phosphorylation at Tyr1020 may be to allow association with SHC and DOK1 (Tridandapani et al., 1997; Tamir et al., 2000), however it is unclear how these associations can affect the membrane association or catalytic activity of SHIP. On the other hand, the role of specific interactions in promoting adaptor functions of SHIP have been described (Tridandapani et al., 1997; Tamir et al., 2000). Moreover, phosphorylation of Tyr1020 in response to long-term IL-4 stimulation has been shown to promote proteasomal degradation of SHIP (Ruschmann et al., 2010).

Contrary to tyrosine phosphorylation, phosphorylation on serine 440 in the phosphatase domain of SHIP by protein kinase A can directly enhance catalytic activity in B cells (Zhang et al., 2009a, 2010a). A recently described model proposes that the SHIP SH2 domain can bind its tyrosine-phosphorylated C-terminal tail potentially generating an auto-inhibited conformation. This implies that other phosphopeptide motifs must compete with the SHIP C-terminus for binding to the SHIP SH2 domain (Mukherjee et al., 2012). Another proposed regulatory mechanism is allosteric activation induced by PI(3,4)P2 binding through SHIP’s C2 domain (Ong et al., 2007). This model suggests a potential “feed-forward” activation of SHIP by its product that can be exploited by SHIP-activating compounds (Ong et al., 2007). All of these regulatory mechanisms, including control of protein expression, localization, phosphorylation on tyrosine and serine, and conformational state, likely contribute to SHIP function in B cells.

### PHOSPHOINOSITIDE DYNAMICS AND REGULATION BY PI PHOSPHATASES

PI3K activation through BCR ligation leads to a transient increase in PI(3,4,5)P_3_ (PIP_3_) and PI(3,4)P_2_ in B cell lines (Gold and Aebersold, 1994; Fruman and Bismuth, 2009). PIP_3_ levels peak after approximately 1 min and return to baseline after 10 min in primary mouse B cells (Clayton et al., 2002). The PI3K reaction producing PIP_3_ can be directly antagonized by PTEN (Maehama and Dixon, 1998; Anzelon et al., 2003). Later, it was confirmed that PI(3,4)P_2_ can be generated either from direct phosphorylation of PI(4)P by PI3K or from dephosphorylation of PIP_3_ by SHIP (Brauweiler et al., 2000). Brauweiler et al. (2000) showed that PIP_3_ is transiently produced after BCR ligation while PI(3,4)P_2_ levels increase steadily over a much longer period of time, consistent with earlier studies in B cell lines (Gold and Aebersold, 1994). In SHIP-deficient B cells, however, PIP_3_ levels are dramatically increased and sustained while PI(3,4)P_2_ production is significantly abrogated (Brauweiler et al., 2000). In the human BJAB B cell line we found that BCR-induced PIP_3_ responses are rapid and transient, while PI(3,4)P_2_ responses are relatively delayed and sustained (Marshall et al., 2002) and verified that stimulation of B cell lines under conditions that promote SHIP activity results in reduced PIP_3_ levels and a dampening of downstream pathways dependent on PIP_3_ (Krahn et al., 2004).

Although the action of both SHIP and PTEN antagonizes PI3K signaling by consuming PIP_3_, the SHIP product PI(3,4)P_2_ is itself a second messenger subject to further regulation by inositol 4′-phosphatases (Norris and Majerus, 1994; Gewinner et al., 2009). While PTEN can also efficiently dephosphorylate PI(3,4)P_2_ *in vitro*, limited evidence exists for PTEN regulation of PI(3,4)P_2_ levels *in vivo*. Stimulation of cells with H_2_O_2_, which inactivates PTEN and 4-phosphatases but not SHIP (Leslie et al., 2003; Ross et al., 2007), leads to a selective accumulation of PI(3,4)P_2_ (Van der Kaay et al., 1999). Thus differential regulation of PIP_3_ and PI(3,4)P_2_ dynamics under different stimulation conditions may specify distinct cellular outcomes based on recruitment of differentially regulated effector molecules.

### PIP_3_ AND PI(3,4)P_2_ BINDING PROTEINS

Phosphoinositides generated by active PI3Ks provide binding sites for some signaling proteins containing PI-binding domains such as PH domains (Dowler et al., 2000; Lemmon and Ferguson, 2000) and phox homology (PX) domains (Kanai et al., 2001; Sato et al., 2001). Akt phosphorylation is often used as a readout of PI3K activity, as D3 phosphoinositides are required to recruit Akt itself to the cell membrane along with its activator, PDK (Burgering and Coffer, 1995; Anderson et al., 1998). The PH domain of Akt binds to both PIP_3_ and PI(3,4)P_2_ (Frech et al., 1997). While PI(3,4)P_2_ has been implicated in Akt phosphorylation and activation (Frech et al., 1997; Ma et al., 2008) some studies suggest that PIP_3_ is the limiting factor for Akt membrane recruitment and activation (Astoul et al., 1999). Notably, Akt activation appears to be increased by selective deregulation of PIP_3_ via loss of SHIP (Liu et al., 1998) or selective deregulation of PI(3,4)P_2_ by loss of INPP4 (Gewinner et al., 2009). Thus Akt phosphorylation may integrate to some extent both PIP_3_ and PI(3,4)P_2_ levels. Direct evidence for the function of Akt in BCR signaling is scarce, however a recent study reported that combined deletion of Akt1 and Akt2 in B cells affects B cell maturation and survival (Calamito et al., 2010).

Another key process triggered by BCR-induced PIP_3_ production is the formation of a “signalosome” involving the tyrosine kinase Btk which contributes to PLCγ2 activation and Ca^2+^ flux (O’Rourke et al., 1998; DeFranco, 2001; Engels et al., 2001; Chiu et al., 2002). Btk contains a PH domain that binds to PIP_3_ with a high degree of selectively (Salim et al., 1996), and loss of PIP_3_ binding leads to loss of Btk function (Rawlings et al., 1993). BCR stimulation leads to a rapid, transient rise in PIP_3_ levels that temporally correlates with the membrane recruitment of Btk (Marshall et al., 2002). Both PIP_3_ levels and Btk PH recruitment can be inhibited by PI3K inhibition or by conditions that promote SHIP activity (Krahn et al., 2004). Bypassing PI3K-dependent recruitment of Btk by a membrane targeted Btk construct overcomes the inhibitory effect of SHIP on Ca^2+^ flux (Bolland et al., 1998).

B cells also express a number of adaptor proteins and guanine nucleotide exchange factors that contain PI-binding domains with various degrees of selectivity for PI3K products. The PH domain adaptor protein Bam32 is selectively expressed in immune cells and has high affinity and high selectivity for binding to PIP_3_ and PI(3,4)P_2_, while TAPP adaptor proteins selectively bind PI(3,4)P_2_ (reviewed in Zhang et al., 2009b). TAPP adaptors show delayed and sustained membrane localization after BCR stimulation which correlated well with the timing of the PIP_3_ to PI(3,4)P_2_ conversion (Marshall et al., 2002; Krahn et al., 2004). H_2_O_2_ induced selective recruitment of TAPP proteins in B cells (Cheung et al., 2007). Membrane recruitment of TAPP1-PH is significantly impaired when PTEN is re-expressed in PTEN-null B cells (Cheung et al., 2007) or when inositol 4′-phosphatase is over-expressed in non-immune cells (Ivetac et al., 2009), suggesting that PI(3,4)P_2_-dependent responses can be regulated by both of these phosphatases. Given the differential control of PIP_3_ and PI(3,4)P_2_ by phosphatases and the existence of distinct binding proteins, it seems likely that these phosphoinositides impact different aspects of B cell activation.

## ROLES OF THE PI3K PATHWAY IN NORMAL B CELL FUNCTION

### B CELL DEVELOPMENT

B cell development and maintenance are dependent on “positive selection” signals initiated by the pre-BCR and BCR and the PI3K pathway has clearly been demonstrated to be a critical component of these developmental and homeostatic signals. We will provide only a brief overview of this area since excellent reviews covering this in detail are available (Okkenhaug and Fruman, 2010).

Mutations targeting p85α or p110δ have been shown to retard B cell maturation at the pro-B cell stage within the bone marrow and lead to a reduction in mature B cells within the spleen and lymph nodes (Fruman et al., 1999; Suzuki et al., 1999; Clayton et al., 2002; Jou et al., 2002; Okkenhaug et al., 2002). Disruption of BCR-induced PI3K activation in CD19/BCAP double knock-out mice also led to severe impairment in the generation of immature and mature B cell subsets within the spleen and bone marrow (Aiba et al., 2008). In contrast, deletion of p110γ did not affect B cell development; however it did have a profound impact on thymocyte development and mature T cell activation (Sasaki et al., 2000). It was found that combined deletion of p110δ and p110γ impairs B cell development to a greater extent than p110δ deficiency alone, however it is not clear to what extent this reflects a B cell-intrinsic requirement for p110γ (Beer-Hammer et al., 2010). While deficiency in p110α did not affect B cell development or BCR signaling, combined deletion of p110α and p110δ led to a nearly complete block in B cell development (Ramadani et al., 2010). This result suggests that p110α may make an important contribution to tonic pre-BCR and BCR signaling, but is less important for signaling induced by acute BCR cross-linking. p110β appears to be dispensable for both B cell development and activation (Ramadani et al., 2010).

At the pro/pre-B cell transition, PI3K signaling appears to be essential to turn off recombination-activating gene (RAG) expression to ensure allelic exclusion (Tze et al., 2005; Verkoczy et al., 2007). RAG expression must again be down-regulated once B cells reach the immature stage and this process has been shown to specifically depend on p110δ (Llorian et al., 2007). The mechanism for PI3K regulation of RAG expression depends on Akt, which phosphorylates and inactivates the transcription factor FOXO1. FOXO1 is able to directly bind to RAG gene promoters, facilitating the transcription of both RAG1 and RAG2; thus, pre-BCR-dependent PI3K signaling turns off RAG expression by inactivating FOXO1 (Amin and Schlissel, 2008). FOXO1 is however required for early B cell development, since FOXO1 deficiency leads to impaired expression of IL-7Rα and RAG in pro-B cells (Dengler et al., 2008). These results point to dual roles of PI3K signaling in promoting and repressing different aspects of B cell differentiation, which is also observed in late B cell differentiation in germinal centers (GC; see below).

Studies in mice with SHIP deletion suggest that deregulation of PIP_3_ levels can impact B cell maturation. SHIP-deficient mice had decreased numbers of pre- and immature B cells but increased number of total B cells in the spleen. Further study provided evidence for accelerated B cell development and quicker emigration from the bone marrow in the deficient cells, implying that SHIP restrains developing B cells from moving through immature and transitional stages to the mature stage (Brauweiler et al., 2000).

### INNATE-LIKE B CELL POPULATIONS

The PI3K signaling pathway appears particularly critical for generation and/or maintenance of innate-like B cell populations (B1 and marginal zone B cells). B1 and MZ populations are nearly absent in p85- or p110δ-deficient mice (Clayton et al., 2002; Donahue et al., 2004). Subsequent studies found that B cell specific deletion of p110δ led to loss of both MZ and B1 B cells, indicating an intrinsic requirement for PI3K in the development of these cells (Rolf et al., 2010). In contrast, follicular B cells numbers are not markedly diminished in p110δ deficient mice; however dual loss of p110δ and CD19 does substantially reduce FO B cell numbers (Kovesdi et al., 2010), suggesting other p110 isoforms and/or other pathways activated by CD19 are sufficient to maintain FO B cells in the absence of p110δ. p110δ mutant mice also showed reduced natural antibodies and reduced T-independent responses characteristic of innate-like B cell populations (Okkenhaug et al., 2002; Durand et al., 2009). Conversely, B1 and MZ populations were increased in mice with B cell specific deletion of PTEN (Anzelon et al., 2003). Combined deficiency of p110δ and PTEN restored the B1 population to normal numbers, but only partially reduced the MZ B cell population, suggesting that additional PI3K isoforms may promote MZ B cell development (Janas et al., 2008).

Interestingly, SHIP-deficient mice show a defect in MZ B cell development; however this was reported to be secondary to a defect in marginal zone macrophages rather than a B cell-intrinsic defect (Karlsson et al., 2003). This suggests that increased PIP_3_ levels may not be sufficient for innate-like B cell expansion: deregulation of both PIP_3_ and PI(3,4)P_2_ (i.e., PTEN deficiency) may be required. The effect of B cell specific deletion of SHIP on innate-like B cell development has not been reported to our knowledge. Combined deficiency of Akt1 and Akt2 led to selective reduction in B1 and MZ populations (Calamito et al., 2010), indicating that Akt is one of the PI3K effectors required for innate-like B cell development. The FOXO1 transcription factor, which is a direct target of Akt, is also implicated in MZ B cell development (Chen et al., 2010).

Several studies indicate that the PI3K pathway is also involved in both homeostatic maintenance and activation of innate-like B cell subsets. Treatment of normal mice with the p110δ inhibitor IC87114 leads to a striking depletion of MZ B cells from the spleen (Durand et al., 2009), suggesting that continuous PI3K tonic signals may be required to maintain the MZ compartment. Consistent with this study, we have found that oral treatment with IC87114 leads to significant MZ B cell depletion within 4 days and appears to be reversible upon discontinuation of treatment. Since integrin blockade was also found to deplete MZ B cells from the spleen (Lu and Cyster, 2002), it is possible that acute p110δ inhibition may impact on the MZ B cell compartment by antagonizing the adhesive interactions of MZ B cell within this microenvironment. This would seem to parallel the findings in B cell leukemia where acute p110δ inhibition leads to release of leukemic cells from lymphoid tissues into circulation (see below). However more work will be required to determine the contributions of B cell-intrinsic de-adhesion versus indirect effects of p110δ inhibition on the microenvironment and/or induction of apoptosis due to loss of tonic BCR signaling.

p110δ mutation or pharmacological blockade inhibited the ability of splenic B cells to produce IL-10 after stimulation with TLR ligands (Dil and Marshall, 2009), consistent with findings that marginal zone B cells are a major source of IL-10 and may have a unique capacity to generate regulatory B cells (Lund, 2008). p110δ blockade also impairs TLR-induced proliferation and chemokine-induced adhesion and migration responses of MZ B cells *in vitro* (Durand et al., 2009). The latter findings contradict the paradigm which suggests that only class 1B PI3Ks are linked to G protein-coupled receptors such as chemokine receptors; however they are consistent with findings in neutrophils which clearly show that p110δ functions in migration responses and respiratory burst triggered by GPCRs (Sadhu et al., 2003; Condliffe et al., 2005).

### ANTIGEN PRESENTATION

**FIGURE 2:**
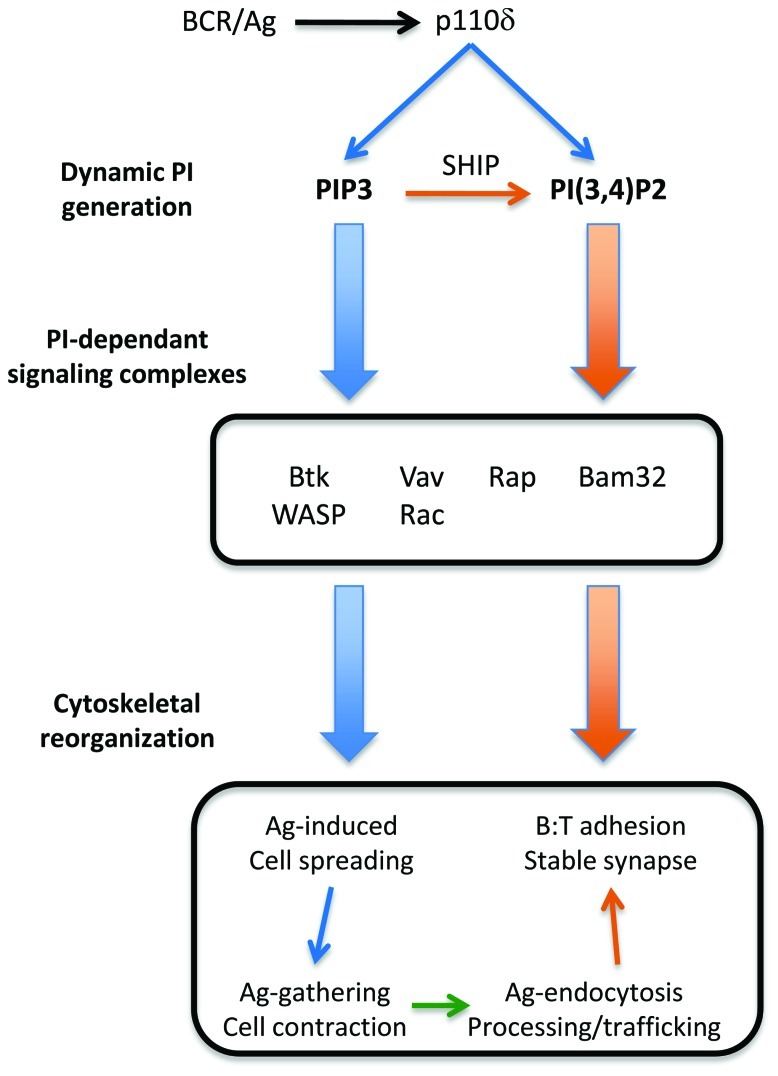
**Role of the PI3K pathway in B cell antigen presentation functions**. The Figure outlines the D3 phosphoinositides and PI-dependant signaling complexes implicated in cytoskeletal reorganization associated with B cell Ag uptake and presentation. The involvement of different components in different steps of Ag presentation is discussed in the text.

B cell Ag presentation involves a number of complex cell biological processes, including BCR–Ag endocytosis and intracellular trafficking, formation of specialized compartments for Ag degradation and peptide loading onto MHC molecules, and formation of stable cell–cell conjugates with Ag-binding T cells (**Figure 2**). Several early studies using high doses of less specific PI3K inhibitors found that PI3K activity was required for optimal B cell Ag presentation function *in vitro* (Granboulan et al., 2003). Some studies suggested PI3K-dependent signal are required at the level of BCR endocytosis (Phee et al., 2001); however we and others have found that BCR endocytosis of soluble Ag does not require PI3K (Al-Alwan et al., 2007). Studies using Ags tethered to artificial membranes found that Ag uptake is associated with a cell spreading and contraction response that requires p110δ PI3K, CD19, Vav, and Rac (Arana et al., 2008; Depoil et al., 2008). These studies suggest that physiological “Ag gathering” from cell surfaces likely requires 3-phosphoinositide-dependent activation of Rac GTPases (**Figure 2**).

We found that pharmacological or genetic inactivation of p110δ impairs BCR-mediated Ag presentation function, and this was associated with a defect in generation of polarized conjugates with cognate T cells (Al-Alwan et al., 2007). It remains to be determined whether PI3K-dependant signals impact intracellular Ag trafficking or generation of the MHCII peptide loading compartment; however we found no effect of PI3K inhibitors on generation of the MHCII–peptide complexes in a murine cell line. On the other hand, p110δ is clearly required for efficient B cell adhesion mediated by LFA1–ICAM and chemokine-induced cell spreading and migration (Durand et al., 2009). In contrast with p110δ inhibition, p110δ deletion was not found to impair BCR-mediated Ag presentation (Rolf et al., 2010). The reasons for this discrepancy are unclear, but may reflect compensatory mechanisms in the deletion model, or other differences in systems used (e.g., different BCR expression/affinity in transgenic versus non-transgenic systems). The SLAM family receptor CD150, known to have a selective role in B:T cell adhesion (Cannons et al., 2010), was shown to activate the PI3K pathway (Mikhalap et al., 2004), suggesting another possible PI3K-dependant adhesion mechanism. Together these studies suggest that PI3K signaling may primarily impact B:T conjugate formation at the level of cell migration and adhesion.

A few PI-binding proteins have been implicated in B cell migration, adhesion, and cell:cell conjugate formation (**Figure 2**), however the mechanisms remain incompletely understood. The Rac-GEF Vav, which has a PIP_3_-binding PH domain, is important in B cell spreading and contraction responses (Arana et al., 2008). We found that the Bam32/DAPP1 adaptor promotes efficient Rac activation and B cell spreading on integrin ligands (Al-Alwan et al., 2010). The activation of Rap GTPase, which is important for cytoskeletal rearrangements occurring in B cell adhesion, spreading, and migration responses (Lin et al., 2008), was found to be dependent on p110δ (Durand et al., 2009).

While the relative importance of PIP_3_ and PI(3,4)P_2_ in B cell Ag gathering and conjugate formation is not clear, SHIP appears to play both positive and negative roles. SHIP-deficient B cells show increased spreading responses and increased F-actin accumulation, presumably due to increased Btk recruitment, but impaired centripetal movement and growth of BCR clusters (Liu et al., 2011). This study suggests that while PIP_3_ drives Btk-dependent F-actin polymerization required for Ag-induced B cell spreading, conversion to PI(3,4)P_2_ by SHIP may then promote “gathering” of BCR–Ag microclusters into large aggregates for internalization. In other cell systems, SHIP has been found to promote certain cellular responses related to cytoskeletal dynamics (Nishio et al., 2007; Severin et al., 2007; Harris et al., 2011). For example, although knock-down of SHIP in primary human T cells resulted in increased total F-actin, it also paradoxically led to loss of actin-rich microvilli projections (Harris et al., 2011). Although incompletely understood, recruitment of PI(3,4)P_2_-specific effectors by SHIP such as TAPP2 (Dowler et al., 2000) and lamellipodin (Krause et al., 2004) may be central to these and other positive regulatory mechanisms.

### CLASS SWITCH RECOMBINATION

We and others have found that PI3K regulates Ig class switch recombination (CSR), with either pharmaceutical blockade or genetic deficiencies in PI3K leading to markedly enhanced switch to IgG1 or IgE isotypes (Omori et al., 2006; Zhang et al., 2008). CSR, also known as isotype switching, is a specific DNA recombination mechanism that replaces the currently expressed immunoglobulin heavy chain constant region gene (C_H_) with one downstream C_H_ gene. Switch (S) regions are highly repetitive sequences upstream of each C_H_ gene which are regulated by sterile germline transcripts (GLTs). Specific GLTs are induced by cytokines and T cell-derived signals, such as IL-4Ra and CD40 (Stavnezer et al., 1988; Nambu et al., 2003). In addition, these signals induce the expression of the enzyme AID, which serves a critical catalytic function for CSR.

**FIGURE 3:**
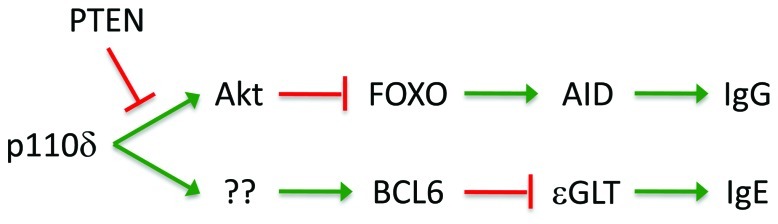
**Role of the PI3K pathway in Ig isotype switch**. The figure depicts the regulatory linkages through which PI3K impacts on Ig isotype switch to IgG and IgE. PI3K-dependent activation of Akt leads to phosphorylation and inactivation of FOXO transcription factors, which drive expression of AID, an enzyme required for all isotype switch. PI3K-dependent, Akt-independent enhancement of BCL6 expression (through an unknown mechanism) promotes the GC B cell gene expression program and also suppresses germline transcription of the IgE locus (eGLT), selectively repressing switch to IgE.

Several lines of evidence indicate that PI3K signaling can suppress CSR through Akt-dependant inactivation of FOXO transcription factors which drive expression of AID (**Figure 3**). PI3K blockade markedly increased expression of AID and this was reversed by constitutively active Akt (Omori et al., 2006). Moreover, we found that inhibition of Akt activity is sufficient to deregulate AID expression (Zhang et al., 2012). Conversely PTEN deficiency leads to reduced isotype switch associated with reduced AID expression (Suzuki et al., 2003). Constitutively active FOXO1 activated AID transcription (Omori et al., 2006) while FOXO1-deficient B cells showed reduced AID expression (Dengler et al., 2008), indicating that Akt regulates AID by inactivating FOXO. While IL-4 is an important driver of CSR, mice with a targeted mutation in the insulin receptor substrate-2 binding site of the IL-4Ra chain, which impairs IL-4 induced PI3K activation, were reported to paradoxically show increased CSR to the IgE isotype (Blaeser et al., 2003). This suggests that PI3K activation via the IL-4R may modulate CSR *in vivo*.

We found that PI3K blockade has a particularly potent effect on CSR to the IgE isotype. Genetic or pharmaceutical inactivation of p110δ resulted in markedly increased IgE levels *in vivo* despite reduced GC responses and reduced production of IL-4 by T cells (Nashed et al., 2007; Zhang et al., 2008). Increased IgE switch was associated with elevated epsilon GLTs (εGLTs) in addition to increased AID expression (Zhang et al., 2008). Production of εGLTs is controlled by the promoter of Iε exon, which contains binding sites for many transcription factors such as STAT6, BCL6, NF-κB, PU.1, PAX5, AP1, and E-box binding sites (Oettgen and Geha, 2001). All of these transcription factors promote the transcription of εGLTs except BCL6, which is a negative regulator that competes with STAT6 for promoter binding (Harris et al., 1999). We recently found that blockade of p110δ PI3K decreases the expression of BCL6, providing a potential mechanism for PI3K regulation of IgE (**Figure 3**). Re-expression of BCL6 was sufficient to reverse excess εGLT expression and IgE switch (Zhang et al., 2012). Interestingly, selective blockade of Akt markedly enhanced AID expression as expected, but had no effect on BCL6 or εGLT expression (Zhang et al., 2012), suggesting other PI3K-dependent signals may regulate BCL6.

### T CELL-DEPENDANT ANTIBODY RESPONSES

The T cell-dependent (TD) antibody response and associated B cell selection in GC is a fascinating system for generation of high affinity isotype-switched antibodies as well as B cell memory. The PI3K pathway acts at multiple levels to regulate these responses generated by follicular B cells. Inactivation of p85 or p110δ or treatment with p110δ-selective PI3K inhibitors significantly perturb functional responses of follicular B cells *in vitro*, including reduced BCR-induced proliferation, increased susceptibility to apoptosis, and impaired adhesion and migration responses (Clayton et al., 2002; Okkenhaug et al., 2002; Durand et al., 2009). It should be noted that some responses are not decreased by p110δ blockade, including proliferative responses induced by CD40 and isotype switch as described above.

*In vivo*, p85 or p110δ mutant mice show dramatically reduced antibody production after immunization with TD Ags (Suzuki et al., 1999; Clayton et al., 2002; Okkenhaug et al., 2002; Okkenhaug and Vanhaesebroeck, 2003). Impairment of BCR-induced PI3K activation by deletion or mutation of CD19 was also found to significantly impair GC differentiation and Ab responses to TD Ags (Rickert et al., 1995). Dual inactivation of PTEN and CD19 was found to restore the ability to mount GC responses (Anzelon et al., 2003). Deletion of SHIP results in enhanced Ab responses to TD Ags (Helgason et al., 2000). However SHIP deletion affects many immune cell types resulting in severe systemic pathology (Helgason et al., 1998), and the impact of B cell specific deletion of SHIP on TD responses has not been reported to our knowledge.

Consistent with failure to induce TD Ab responses, many of the above studies also found that p110δ deficiency virtually abrogates the generation of GC. Similarly we have found that treatment with IC87114 strongly reduces GC responses, without the associated decreased serum Ig levels seen in p110δ mutant mice (Zhang et al., 2008, 2012). We found that IC87114 treatment of mice with a pre-established GC response significantly reduced the number of GC B cells, suggesting that continuous p110δ signaling is required for GC maintenance since. As discussed above p110δ appears to have an ancillary function in maintaining expression of BCL6, a key transcriptional regulator for the GC B cell genetic program, providing a potential mechanism linking p110δ to GC B cell maintenance.

Given the substantial evidence for a B cell-intrinsic requirement for p110δ, it was surprising when Rolf et al. (2010) found that B cell specific deletion of p110δ did not markedly impair TD Ab or GC responses. This result may suggest that other PI3K isoforms such as p110α can functionally compensate for loss of p110δ in the context of the GC response. Since follicular B cell responses to CD40 and TLR ligands seem to be less dependent on the PI3K pathway, it is possible that, under immunization conditions generating abundant T cell help and other adjuvant-induced activation signals, the D3 phosphoinositide levels may not be a limiting factor controlling GC size. Interestingly, Rolf et al. (2010) also found compelling evidence that p110δ plays an important role in follicular helper T cell (T_FH_) function. Thus, the dramatic loss of GC responses associated with p110δ blockade is likely due to the combined impact on GC B cells and T_FH_ cells.

Accumulating evidence indicates that selection of high affinity B cell clones within the GC is driven largely by cognate interactions with T_FH_, which deliver co-stimulatory signals required for continued GC B cell survival (King et al., 2008). Given the evidence outlined above regarding the roles of the PI3K pathway in B cell migration, adhesion and Ag presentation to T cells, PI3K signaling would be expected to have an important role in affinity maturation. However, there is currently limited evidence supporting this proposition. As noted above, p110δ-deficient B cells show only subtle reductions in affinity maturation (Rolf et al., 2010) while p110α/δ double deficiency abrogates B cell development (Ramadani et al., 2010), precluding affinity maturation studies.

We found that the PI-binding adaptor protein Bam32/DAPP1 is required for optimal B cell Ag presentation, and Bam32-deficient mice showed premature dissolution of the GC response and impaired affinity maturation (Zhang et al., 2010b). It should be noted that the GC defect in Bam32-deficient mice was observed under conditions of relatively low precursor frequency of Ag-specific B and T cells (i.e., no Ag receptor transgenes) and with relative mild adjuvant conditions (OVA/alum). Bam32-deficient mice were able to generate relatively normal GC responses after immunization with sheep red blood cells, known to activate large numbers of lymphocytes and to contain a potent adjuvant (hemin). It is tempting to speculate that, under conditions of limiting T cell numbers in GC, BCR-induced activation of PI3K may recruit PI-binding proteins such as Bam32 and Vav to the plasma membrane and provide a selective advantage to GC B cells by enhancing their ability to form cognate interactions. In this context it would be interesting to examine the ability of p110δ-deficient B cells to compete with wild-type B cells within a GC response.

### CONTROL OF B CELL HOMEOSTASIS AND ANERGY

Three studies performed in mice harboring B cell-targeted deletions of PTEN have shed light on the specific roles of this phosphatase in B cell homeostasis (Anzelon et al., 2003; Suzuki et al., 2003; Browne et al., 2009). Both immature and mature PTEN-deficient B cells exhibited hyper-activation and hyper-proliferation in response to various stimuli (Anzelon et al., 2003; Suzuki et al., 2003; Browne et al., 2009). Mature B cell populations also displayed enhanced migration and resistance to apoptotic signals (Suzuki et al., 2003). Two of the studies report an increase in autoantibody titer (Suzuki et al., 2003; Browne et al., 2009), which one study attributed to abnormal generation of innate-like B cells (Suzuki et al., 2003). In the context of chronic self-Ag stimulation, anergic BCR transgenic B cells showed increased expression of PTEN and showed reduced PIP_3_ levels after BCR cross-linking, suggesting that dampening of the PI3K pathway via PTEN may be a significant component of anergy induction (Browne et al., 2009). Consistent with this idea, PTEN-deficient BCR transgenic cells failed to develop anergy in presence of self-Ag (Browne et al., 2009). Notably, PTEN can also act as a protein phosphatase (Myers et al., 1997). The extent to which the effect of PTEN on B cell anergy is due its PI-hydrolyzing function is unclear, and the relative contributions of innate-like B cell populations and GC defects to autoimmunity development remain to be determined.

Deregulation of PI dynamics via deletion of SHIP has also been reported to impact B cell anergy. SHIP-deficient B cells were shown to exhibit heightened *in vitro* responsiveness, including increased phosphorylation of Akt and MAPKs as well as enhanced proliferation, survival and cell cycling upon stimulation through the BCR (Helgason et al., 2000). SHIP^–/–^ mice also display elevated serum Ig levels and increased Ag-specific IgG in response to a T cell-independent Ag (Helgason et al., 2000). Another group found that SHIP-deficient B cells were more sensitive to induction of CD86 expression upon BCR ligation and more sensitive to BCR-induced apoptosis (Brauweiler et al., 2000). Recently, B cell-targeted SHIP-deficient mice were characterized (O’Neill et al., 2011). These mice displayed a severe lupus-like autoimmune phenotype featuring increased autoantibodies produced against nuclear components and IgG deposition in glomeruli of the kidneys. The authors also showed that SHIP phosphorylation is increased in primary anergic B cells from wild-type mice, suggesting that increased SHIP activity may contribute to low PIP_3_ levels. The authors hypothesize that chronic monophosphorylation of Igα/β (CD79a/b) ITAMs by Src family kinases in anergic B cells leads to constitutive activation of SHIP, which is essential for the maintenance of B cell anergy (O’Neill et al., 2011).

We have recently completed a study on mice bearing mutations in the PH domains of TAPP1 and TAPP2 (Landego et al., 2012). These mutations effectively uncouple TAPP adaptors from the SHIP product PI(3,4)P_2_ (Wullschleger et al., 2011). It was found that TAPP mutant B cells display exaggerated proliferative responses to BCR cross-linking which was associated with increased Akt phosphorylation. Strikingly these mice show several additional similarities to SHIP-deficient mice, including increased basal Ig levels, autoantibodies and development of lupus-like disease. Given the similar phenotypes observed in TAPP KI and SHIP-deficient mice, it is tempting to speculate that TAPP-PI(3,4)P_2_ interactions may in part mediate the regulatory effects of SHIP in B cells. The mechanism by which TAPPs antagonize Akt activity is currently unclear, but may involve competition for PI(3,4)P_2_ or recruitment of a regulatory phosphatase.

Consistent with the reported role of PIP_3_ phosphatases in maintaining anergy, haploinsufficiency of PI3K p110δ was very recently reported to partially attenuate the autoimmune phenotype of Lyn-deficient mice (Maxwell et al., 2012). Plasma cell numbers were reduced, as were titers of antibodies and autoantibodies in the serum. The hyper-proliferative B cell phenotype was also moderated. Lyn^–/–^p110δ^+/KD^ B cells maintained high basal and BCR-stimulated Akt and MAPK phosphorylation as well as increased surface expression of CD80 and CD86 characteristic of Lyn-deficient B cells. Since myeloid cells and T cells likely contribute to the reduction of disease severity (Maxwell et al., 2012), the contribution of B cell-intrinsic p110δ signaling in this autoimmune mouse model is not yet clearly defined.

## MECHANISMS OF PI3K ACTIVATION IN MALIGNANT B CELLS

**FIGURE 4:**
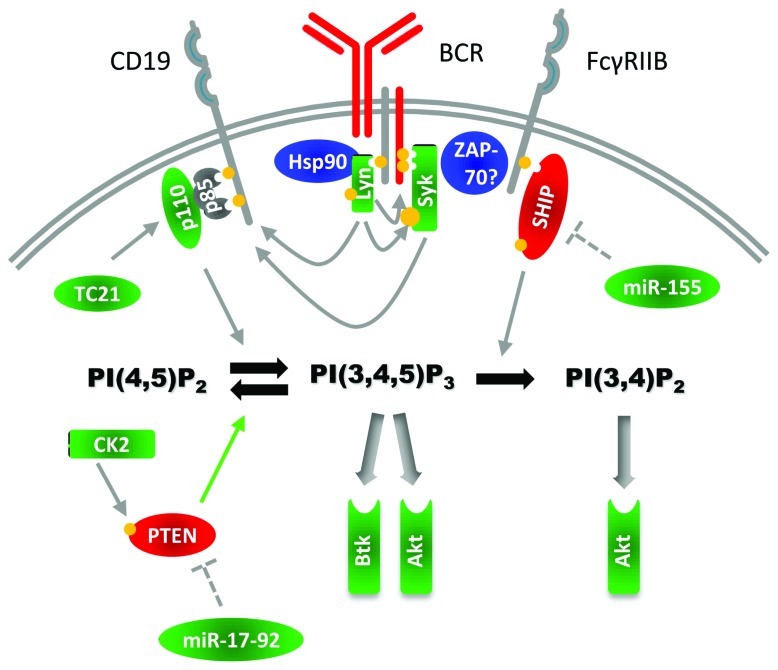
**Alterations in the PI3K pathway in malignant B cells**. In malignant B cells, several pathway players have been found to be over-expressed or hyper-phosphorylated. The figure illustrates signaling molecules whose expression, phosphorylation or activity are reported to be altered in malignant B cells (green indicates increased activity, red indicates reduced activity). Altered molecules discussed in the text include p110 subunits, Lyn, Syk, Btk, Akt, TC21, and the BCR signaling chain CD79b. The formation of a non-physiological complex between Lyn and Hsp90 in malignant B cells is indicated. Increased expression of microRNA species that target PTEN and SHIP are reported to reduce expression of these phosphatases. Moreover, the PTEN kinase CK2 is reported to be over-expressed which may diminish PTEN activity. ZAP-70 is expressed in some B cell malignancies, however the impact on the PI3K pathway is currently not clear.

The finding that BCR signaling via PI3K is critical for mature B cell homeostasis and function has driven interest in understanding the role of the PI3K pathway in malignant B cells. The PIK3CA gene is mutated in many cancers but rarely in hematological disorders, as a recent study on multiple myeloma (MM) confirmed (Ismail et al., 2010). Together with the finding that PTEN mutations are relatively rare in B cell malignancies (Leupin et al., 2003; Georgakis et al., 2006), the initial conclusion was that PI3K signaling was less critical in these diseases compared to solid tumors. However, constitutive PI3K activity is significantly increased in chronic lymphocytic leukemia (CLL) relative to normal B cells (Herman et al., 2010; Ringshausen et al., 2002). Enhanced basal and stimulated Akt phosphorylation is observed in a subset of CLL patients, with increased phosphorylation associated with progressive disease (Barragan et al., 2006; Longo et al., 2007) and decreased phosphorylation associated with an “anergic” phenotype (Muzio et al., 2008). p110δ was reported to be marginally over-expressed in CLL B cells (B-CLL) compared to normal B cells (Herman et al., 2010), and as discussed below, recent results indicate that this isoform is functionally important for Akt activation and survival of malignant B cells. Current findings collectively indicate that elevated PI3K pathway activity in B cell malignancies is driven by altered BCR signaling (**Figure 4**) together with other co-stimulatory signals present in lymphoid tissues such as chemokines and cytokines. Below we review recent studies that identify molecular alterations in malignant B cells affecting the PI3K signaling pathway. We focus particularly on CLL, where there is the most information available and promising clinical results of PI3K inhibitors have been reported in early trials.

### BCR AND ASSOCIATED PROTEIN TYROSINE KINASES

Many B cell malignancies show evidence of altered BCR signaling (**Figure 4**), which is likely relevant to increased cell survival and accumulation in blood and tissues. Compared to normal B cells, B-CLL exhibit low surface expression of Ig and CD79b, associated with low levels of mRNA encoding for CD79b (Thompson et al., 1997). CD79b mRNA level was however found to be higher in the more severe CLL prognostic group associated with unmutated Ig heavy chain variable (V_H_) genes (Cajiao et al., 2007), suggesting a possible link between CD79b expression and disease severity. Some authors have compared this BCR-low phenotype to “anergic” B cells which, in animal models, down-modulate surface BCR in response to chronic BCR stimulation by low affinity or soluble self-Ags. Despite low BCR expression, B-CLL show evidence of constitutively elevated PI3K pathway activity that is dependent on protein kinases normally activated by the BCR, including Lyn and Syk.

Lyn is over-expressed in CLL (Contri et al., 2005; Trentin et al., 2008) and Hodgkin lymphoma (HL; Martin et al., 2011) and is anomalously present in the cytosol where it forms an aberrant complex with Hsp90 (Contri et al., 2005; Trentin et al., 2008). In contrast with normal B cells, Lyn’s activity does not appear to change with anti-IgM stimulation; however Lyn inhibition induced apoptosis in CLL (Contri et al., 2005). Although no Syk mutations were found in CLL (Brown et al., 2008; Philippen et al., 2010), Syk was found to be over-expressed at the protein level (Buchner et al., 2009). In HL, Syk expression was associated with shorter failure-free survival (Martin et al., 2011), while in plasma cell-like Waldenström’s macroglobulinemia, BLNK and Syk genes were reported to be up-regulated (Gutierrez et al., 2007). Constitutive phosphorylation and activation of Syk has been observed in acute lymphocytic leukemia (ALL), follicular lymphoma (FL), diffuse large B cell lymphoma (DLBCL), and CLL, as reviewed elsewhere (Efremov and Laurenti, 2011). The mechanisms underlying aberrant expression and activity of these kinases remain unknown.

A subset of B-CLL can also express the Syk-family kinase ZAP-70, which has been proposed to influence BCR signaling. Although its role in leukemic B cells is not fully understood, ZAP-70 expression is used as a clinical marker predictive of aggressive clinical course (Crespo et al., 2003; Orchard et al., 2004; Rassenti et al., 2004). While ZAP-70 activation through the BCR is inefficient in CLL and lymphoma cell lines and appears to be negligible compared to Syk activation, ZAP-70 can still recruit downstream signaling molecules following BCR stimulation (Gobessi et al., 2007). B cells from ZAP-70 positive CLL patients expressed more phosphorylated Syk, PLCγ, and BLNK than ZAP-70 negative B cells only after anti-IgM stimulation (Chen et al., 2005). Introduction of ZAP-70 into ZAP-70 negative B cells also enhanced Akt phosphorylation (Gobessi et al., 2007), suggesting the effects on tyrosine kinase activation can leads to elevated PI3K activity. In transducing B-CLL with intact or mutant ZAP-70, Chen et al. (2008) identified ZAP-70 SH2 domain but not its kinase activity as necessary to induce calcium flux after BCR stimulation, suggesting that ZAP-70 can function as an adaptor protein in BCR signaling. Interestingly, ZAP-70 expression was reported to both prolong Syk activation and delay BCR internalization (Gobessi et al., 2007; Calpe et al., 2011), suggesting that it could help reduce BCR down-modulation in response to chronic stimulation by self-Ags.

Besides altered protein tyrosine kinase activity, other mechanisms potentially affecting PI3K pathway activity in malignant B cells have been proposed. The GTPase TC21, which promotes PI3K activity presumably by recruiting p110δ to the BCR, is over-expressed in DLBCL and HL compared to healthy donors (Delgado et al., 2009), providing another potential gain-of-function mechanism. Notably, the relative importance of classical modes of PI3K recruitment downstream of BCR signaling, such as through CD19 or BCAP binding, versus other mechanisms such as TC21- or insulin receptor substrate-dependent recruitment, is unknown in malignant B cells. The adaptor p66Shc, which can inhibit Akt phosphorylation and promote apoptotic signaling via the BCR (Pacini et al., 2004), was profoundly impaired in B-CLL compared to normal B cells, with lower expression in the unfavorable prognosis group (Capitani et al., 2010). The protein tyrosine phosphatase receptor-type O (PTPRO) has been implicated as a significant regulator of protein tyrosine kinase activity in B cell malignancy. Following the identification of Syk as a target for the truncated form of PTPRO (PTPROt) in a BCR stimulation-independent manner (Chen et al., 2006), ZAP-70 and Lyn activities were shown to be inhibited by PTPROt in leukemic cells (Motiwala et al., 2010). In CLL, extensive methylation of the CpG island in the gene encoding PTPROt was detected in 82% of patients (Motiwala et al., 2007). These findings suggest that Lyn, Syk, and ZAP-70 hyper-activity could be due in part to epigenetic silencing of their negative regulator and that this system regulates tonic signaling from BCR.

### PI PHOSPHATASES

Although PTEN is frequently mutated in several kinds of cancers, it is rarely mutated in B cell malignancies (Leupin et al., 2003; Georgakis et al., 2006). PTEN mutations were found in 5% of primary lymphomas (Gronbaek et al., 1998; Sakai et al., 1998) and in two primary effusion lymphoma cell lines but not in primary cells (Boulanger et al., 2009). However PTEN expression and function are regulated at transcriptional and post-transcriptional levels via microRNAs, phosphorylation, ubiquitination and oxidation, and substantial evidence now indicates that malignant B cells frequently alter PTEN protein expression and function through such mechanisms.

Several studies showed a reduction or loss of PTEN expression in DLBCL (Abubaker et al., 2007; Liu et al., 2010) and CLL (Leupin et al., 2003). We found that the BJAB B cell lymphoma has no detectable PTEN protein and highly elevated generation of PIP_3_ and PI(3,4)P_2_ (Marshall et al., 2002; Cheung et al., 2007), however PTEN mRNA appeared to be expressed normally in these cells and contained no mutations. The miR-17-92 has emerged as an important negative regulator of PTEN expression. This microRNA cluster is over-expressed in several leukemias and lymphomas (Lenz et al., 2008; Rao et al., 2011) providing a potential mechanism for PTEN down-regulation. Signaling via NOTCH1 is reported to activate the PI3K pathway by inhibiting PTEN transcription (Palomero et al., 2007) and NOTCH1 was recently found to be mutated in CLL (Rosati et al., 2009; Fabbri et al., 2011; Balatti et al., 2012; Rossi et al., 2012) and is a predictor of survival (Rosati et al., 2009; Del Giudice et al., 2012; Rossi et al., 2012). Moreover, PTEN enzymatic activity is reported to be deficient in CLL (Shehata et al., 2010), suggesting that PTEN post-translational regulation is also altered in hematological cancer. In HL cell lines, reduced PTEN function was suggested to be due to its phosphorylated status (Georgakis et al., 2006). A well-known PTEN regulator CK2 is over-expressed and hyper-activated in CLL and CK2 blockade decreased PTEN phosphorylation, restoring PTEN activity (Shehata et al., 2010; Martins et al., 2011).

Since SHIP is also an important regulator of PI3K signaling, one might expect it to have tumor suppressor properties in the immune cells. Indeed, this has been demonstrated for some hematological malignancies (Fukuda et al., 2005; Vanderwinden et al., 2006). Recent evidence, including data from human samples as well as mouse models and tumor cell lines, supports a tumor suppressor role in certain B cell malignancies as well. SHIP was identified as a target of miR-155 (Costinean et al., 2009; Pedersen et al., 2009), which is over-expressed in several B cell lymphomas (Eis et al., 2005; Kluiver et al., 2005). One group studying DLBCL found that the more aggressive disease type (non-GC DLBCL) was associated with higher levels of miR-155, and consequently lower levels of SHIP, compared to GC DLBCL. Patients with the least SHIP expression also had the worst survival outcome (Pedersen et al., 2009). Another group independently confirmed that SHIP is often down-regulated in DLBCL patients (Miletic et al., 2010). Interestingly, SHIP down-regulation occurs more frequently in patients that have also down-regulated PTEN (Miletic et al., 2010). Moreover, B cells from ZAP-70 positive CLL patients exhibited decreased expression of SHIP as well as decreased SHIP phosphorylation both basally and induced by BCR cross-linking (Gabelloni et al., 2008).

Animal models support a role for SHIP as a tumor suppressor in B cells. Transgenic mice overexpressing miR-155 develop a mixed tumor phenotype with characteristics of ALL and high grade lymphoma (Costinean et al., 2006). Further study revealed that the highest miR-155 transgene expression in these mice occurred in the bone marrow and, specifically, in pre-B cells. The authors identified SHIP as one of the miR-155 targets which showed decreased expression in pre-B lymphocytes and declined further during progression to leukemia. Another mouse model highlighting a protective role for SHIP in B cell cancers is a B cell-specific double knock-out of PTEN and SHIP (Miletic et al., 2010). The authors found that unlike with B cell-specific deletion of either phosphatase, lethal B cell neoplasms arose spontaneously in double-deficient mice. Since SHIP deletion is sufficient to substantially deregulate PIP_3_ levels in B cells, its seems likely that additional functions of PTEN, such as regulation of PI(3,4)P_2_ or protein phosphatase activity may be required to poise B cells for proliferative expansion.

Several studies have explored the impact of small molecule agonists or antagonists of SHIP in B cell malignancies. When MM cell lines were treated with a specific allosteric activator of SHIP, AQX-MN100 (Ong et al., 2007), cell viability was significantly reduced, at least in part due to induction of apoptosis (Kennah et al., 2009). Surprisingly, another group provide evidence that treatment with compounds designed to inhibit rather than activate SHIP reduced tonic and agonist-induced Akt activation and decreased viability of blood cancer cells, including human MM cell lines (Brooks et al., 2010). The authors suggest that these effects reflect that Akt requires PI(3,4)P_2_ in addition to PIP_3_ for full activation (Scheid et al., 2002). This study also provided some *in vivo* evidence of the therapeutic potential of SHIP inhibition using a tumor xenograft model. Interestingly, mice that were resistant to treatment were found to have up-regulated SHIP2 in their tumor cells (Fuhler et al., 2012).

The suggestion that both activation and inhibition of SHIP in MM cells have potential therapeutic benefits is puzzling. Certainly the use of different cell lines is a major limitation in determining the relevance of these studies, and off-target effects of these compounds cannot be ruled out. It is conceivable, however, that both activation and inhibition of SHIP could independently lead to apoptosis by different mechanisms. For example, activation of SHIP leads to reduction of PIP_3_ which helps dampens effector functions including cell proliferation, thus limiting tumor growth. On the other hand, SHIP inhibition could reduce PI(3,4)P_2_ levels, affecting Akt activation or impacting other PI(3,4)P2-binding proteins such as the TAPP proteins. It is also possible that SHIP inhibition leads to chronically elevated PIP_3_ levels which could trigger activation-induced cell death. Future studies in primary human MM cells will hopefully shed light on which approach has the most therapeutic potential and in what disease context.

## PI3K PATHWAY INHIBITION AS A THERAPEUTIC STRATEGY FOR B CELL MALIGNANCIES

As described above, the PI3K pathway plays pivotal roles in B cell responses such as survival, activation, proliferation, cytoskeleton dynamics, migration, and adhesion. Since the PI3K pathway is deregulated at multiple levels in malignancies, this has become a major target for new therapies. In fact, as PI3K inhibitors enter clinical trials, some of the first success stories have come from B cell malignancies. Given that PI3K enzymes do not act as a classical mutated oncogene in these diseases, this early success has come as somewhat of a surprise to many in the field. In this section we will focus on clinical results in CLL, which have taught us much about therapeutic considerations and mechanisms of PI3K inhibitors.

### *IN VITRO* PI3K INHIBITOR STUDIES

The PI3K inhibitors LY294002 and wortmannin have both been shown to have activity against CLL *in vitro*. LY294002 helped to identify the PI3K pathway as the major pathway responsible for IL-4 and plasma albumin-induced protection from apoptosis (Wickremasinghe et al., 2001; Barragan et al., 2002; Jones et al., 2003). Phorbol 12-myristate 13-acetate (PMA)-induced Akt activation is relatively insensitive to LY294002 (Barragan et al., 2006). This PI3K inhibitor was suggested to induce B-CLL apoptosis by reducing X-linked inhibitor of apoptosis protein (XIAP) expression (Ringshausen et al., 2002), caspase 8 cleavage (Plate, 2004), and Mcl-1 expression (Ringshausen et al., 2002; Spagnuolo et al., 2011). Wortmannin was found to inhibit B-CLL migration to stromal cells mediated by CXCR4–CXCL12 (Burger et al., 1999) and CXCR5–CXCL13 (Burkle et al., 2007). Moreover, LY294002 treatment enhanced B-CLL apoptosis induced by Fludarabine (DNA synthesis inhibitor) or Dexamethasone (corticosteroid; Barragan et al., 2002), identifying the PI3K pathway as a good candidate for combination drug therapy. LY294002 and wortmannin failed to enter clinical trials: LY294002 because of dermal toxicity and low bioavailability (Hu et al., 2000); wortmannin, due to liver and hematologic toxicity as well as instability of the molecule (Ihle et al., 2004). Both of these compounds have also been found to have significant off-target effects (Knight et al., 2006).

Recent drug development efforts have focused on generating compounds with improved specificity and bioavailability, as well as targeting specific PI3K catalytic subunits (Marone et al., 2008). Two more recently developed inhibitors, PI-103 and PIK-90, are defined as p110α multi-target inhibitors (Knight et al., 2006), meaning they behave as pan-PI3K pathway inhibitors at high doses (Raynaud et al., 2007) but can be used as p110α-specific inhibitors at low doses (Niedermeier et al., 2009). Our group used PI-103 to study the role of the PI3K pathway in B-CLL adhesion to stromal cells, previously shown to provide efficient B-CLL protection (Kurtova et al., 2009). We observed that PI-103 abrogated B-CLL binding to stromal cells and inhibited B cell survival. PI3K inhibition blocked adhesion of both ZAP-70 positive and ZAP-70 negative B-CLL and reversed enhanced adhesion induced by CD40L + IL-4, IL-6, or IL-8 (Lafarge et al., in preparation). Moreover, PI3K multi-target inhibitors were found to inhibit B-CLL migration toward stromal cells via CXCL12, as well as inhibit Akt and S6 phosphorylation more efficiently than specific p110δ (IC87114) and p110β/δ (TGX115) inhibitors (Niedermeier et al., 2009). These drugs also reversed stromal cell protection and enhanced Fludarabine-induced apoptosis (Niedermeier et al., 2009). These results indicate that PI3K inhibition is a promising strategy to reverse B-CLL protection from apoptosis mediated by stromal cell interactions.

Recent studies also indicate that specific inhibitors of p110δ can have activity against CLL *in vitro*. CAL-101/GS-1101 is a potent, orally bioavailable PI3K inhibitor highly selective for the p110δ isoform. It has been shown to have activity against multiple B cell malignancies (Lannutti et al., 2011). *In vitro*, CAL-101 reduced B-CLL survival, associated with inhibition of Akt (Herman et al., 2010; Hoellenriegel et al., 2011) and ERK (Hoellenriegel et al., 2011) pathways, more efficiently than LY294002 (Herman et al., 2010). CAL-101 was also shown to induce B-CLL apoptosis despite addition of protective factors such as CD40L, BAFF, TNF-α, anti-IgM, fibronectin, nurse-like cells (NLC) co-culture, or stromal cells. Notably, CAL-101 did not affect IL-4-induced survival (Herman et al., 2010; Hoellenriegel et al., 2011), suggesting that IL-4 in this context signals survival through another pathway or another PI3K isoform. In addition to CLL, recent studies suggest CAL-101 may have therapeutic activity in MM (Ikeda et al., 2010) and HL cells (Meadows et al., 2012).

In B-CLL co-cultures with NLC, CAL-101 inhibited the production of many cytokines and chemokines (CCL7, CCL17, CCL22, CXCL13, CD40L, and TNF-α). Interestingly, IL-6 levels were unaltered following CAL-101 treatment in this co-culture system (Hoellenriegel et al., 2011), implying that production of this cytokine alone was p110δ-independent. Moreover, CAL-101 inhibited B-CLL migration toward CXCL12, CXCL13, and stromal cell lines (Hoellenriegel et al., 2011). In normal cells, CAL-101 showed low toxicity for T and NK cells, but significantly reduced production of IFN-γ by NK cells and production of several T cell cytokines (IL-6, IL-10, TNF-α, and CD40L mRNA). Consistent with this finding, we have found that the p110δ inhibitor IC87114 markedly inhibits cytokine production by human T cells, while inducing minimal apoptosis (Lotoski et al., submitted). The production of these protective cytokines was also reduced in NK and T cells from CLL patients (Herman et al., 2010). Lastly, CAL-101 enhanced the cytotoxic activity of Fludarabine, Dexamethasone, and Bendamustine (DNA synthesis inhibitor; Hoellenriegel et al., 2011). Together these results suggest that p110δ inhibition has potential to selectively block both intrinsic (BCR tonic) and extrinsic signals from the lymphoid tissue microenvironment promoting B-CLL survival and proliferation.

### EARLY CLINICAL TRIAL RESULTS

Early clinical trial results have revealed potential efficacy as well as insights into the *in vivo* mechanisms of action of p110δ inhibitors. After 28 days of CAL-101 treatment, plasma from CLL patients showed lower levels of CCL3/4 (Hoellenriegel et al., 2011) and CXCL13 (Brown et al., 2011; Hoellenriegel et al., 2011) and B-CLL from these patients showed lower levels of Akt phosphorylation (Brown et al., 2011; Hoellenriegel et al., 2011). Strikingly, these patients presented with increased numbers of B-CLL in the peripheral blood (absolute lymphocyte count, ALC), presumably reflecting release of the malignant cells from lymphoid tissues (Hoellenriegel et al., 2011). Patients treated with CAL-101 plus Rituximab or Bendamustine did not increase their ALC, potentially reflecting more efficient killing of B-CLL after release from protective tissue microenvironments (Castillo et al., 2012). Brown et al. (2011) reported marked lymph node shrinkage and found that ALC increased shortly after treatment with either CAL-101 alone or combination therapies. With CAL-101 alone, ALC stayed high for an extended period, whereas with CAL-101 plus Bendamustine ALC rapidly decreased over time.

Together, current data suggests that p110δ inhibition releases B-CLL from their protective microenvironment but does not by itself efficiently induce their apoptosis *in vivo*. Since CLL is characterized by the progressive accumulation of B-CLL in the peripheral blood, lymph nodes, spleen, and bone marrow (Cheson et al., 1996; Hallek et al., 2008), and these microenvironments play a major role in providing survival signals from supporting cells, this “tissue release” action seems likely to provide clinical benefit. This finding was somewhat unexpected, however it is consistent with accumulating data from *in vitro* models showing that PI3K inhibitors can disrupt B-CLL-stromal interactions and with mouse models showing that p110δ inhibition can release marginal zone B cells from the spleen. Mechanistically, it is currently unclear whether CAL-101 acts by directly antagonizing B-CLL intrinsic chemotactic/adhesion responses required for tissue retention or by altering production of chemotactic/adhesive factors produced within other lymphoid tissue cells. Clinical trials with CAL-101 and other PI3K inhibitors are on-going and have expanded to other B cell malignancies including MM, NHL, FL, HL, small lymphocytic lymphoma, and acute myeloid lymphoma.

An interesting analogy was observed between CAL-101 and the Btk inhibitor PCI-32765 (Ibrutinib). Briefly, this Btk inhibitor induced apoptosis in B-CLL but not in normal cells (Herman et al., 2011) and reversed the microenvironment-induced survival (Herman et al., 2011), signaling (Herman et al., 2011; de Rooij et al., 2012), adhesion (de Rooij et al., 2012), and migration (Herman et al., 2011; Ponader et al., 2012). Moreover, in an adoptive transfer TCL1 mouse model of CLL, PCI-32765 was shown to slow decrease progression (Chen et al., 2011; Ponader et al., 2012). In early clinical trials, reduced lymph node size and increased ALC were observed in almost all patients, as reviewed in (Ma and Rosen, 2011). It seems that inhibiting p110δ or Btk have similar outcomes: releasing B-CLL from their protective niche, leading to clinical improvement.

## CONCLUDING REMARKS

Studies of PI3Ks and the phosphatases that regulate their products have revealed the complex system of checks and balances that control phosphoinositide accumulation. Clearly B cells can integrate multiple signaling inputs to control the pathway appropriately under various biological circumstances. Interpretation of the literature is complicated by the fact that most functional studies looking at the roles of PI3K, PTEN, or SHIP do not include PI lipid measurements. Given the discovery of various PI phosphatase-independent functions of PTEN and SHIP, further work is needed to verify conclusions about the roles of different PI species in B cell biology. Of particular interest for the future is understanding the signaling mechanisms linked to is the independently regulated PI(3,4)P_2_ and their roles in normal and malignant B cell functions.

The emerging importance PI3K pathway in B cell malignancies seems to derive not from not from classical oncogenic or tumor suppressor mutations, but from more subtle re-wiring of the BCR-linked activation mechanisms present in normal B cells. Thus, understanding the regulation and functions of the pathway in normal B cells will continue to inform studies in B cell malignancy as clinical applications move forward. Despite a significant literature on aberrant PI3K signaling in malignancy, the impact of these changes on PI lipid dynamics remains largely unstudied, representing a major gap in our understanding. Finally, as clinical results have highlighted the importance of external influences on the PI3K pathway such as factors in the lymphoid tissue microenvironment, the contribution of this pathway in cell:cell interactions influencing normal and malignant B cell biology is an important area for future study.

## Conflict of Interest Statement

The authors declare that the research was conducted in the absence of any commercial or financial relationships that could be construed as a potential conflict of interest.
